# The Peutz-Jeghers kinase LKB1 suppresses polyp growth from intestinal cells of a proglucagon-expressing lineage in mice

**DOI:** 10.1242/dmm.014720

**Published:** 2014-09-04

**Authors:** Sagen Zac-Varghese, Stefan Trapp, Paul Richards, Sophie Sayers, Gao Sun, Stephen R. Bloom, Frank Reimann, Fiona M. Gribble, Guy A. Rutter

**Affiliations:** 1 Department of Investigative Medicine, Imperial College London, London, W12 ONN, UK.; 2 Department of Surgery and Cancer, Imperial College London, London, W12 ONN, UK.; 3 Cambridge Institute for Medical Research, University of Cambridge, Hills Road, Cambridge, CB2 0XY, UK.; 4 Department of Cell Biology, Imperial College London, London, W12 ONN, UK.

**Keywords:** Glucagon, LKB1, Peutz-Jeghers

## Abstract

Liver kinase B1 (LKB1; also known as STK11) is a serine/threonine kinase and tumour suppressor that is mutated in Peutz-Jeghers syndrome (PJS), a premalignant syndrome associated with the development of gastrointestinal polyps. Proglucagon-expressing enteroendocrine cells are involved in the control of glucose homeostasis and the regulation of appetite through the secretion of gut hormones such as glucagon-like peptide-1 (GLP-1) and peptide tyrosine tyrosine (PYY). To determine the role of LKB1 in these cells, we bred mice bearing floxed alleles of *Lkb1* against animals carrying Cre recombinase under proglucagon promoter control. These mice (GluLKB1KO) were viable and displayed near-normal growth rates and glucose homeostasis. However, they developed large polyps at the gastro-duodenal junction, and displayed premature mortality (death from 120 days of age). Histological analysis of the polyps demonstrated that they had a PJS-like appearance with an arborising smooth-muscle core. Circulating GLP-1 levels were normal in GluLKB1KO mice and the polyps expressed low levels of the peptide, similar to levels in the neighbouring duodenum. Lineage tracing using a Rosa26tdRFP transgene revealed, unexpectedly, that enterocytes within the polyps were derived from non-proglucagon-expressing precursors, whereas connective tissue was largely derived from proglucagon-expressing precursors. Developmental studies in wild-type mice suggested that a subpopulation of proglucagon-expressing cells undergo epithelial-mesenchymal transition (EMT) to become smooth-muscle-like cells. Thus, it is likely that polyps in the GluLKB1KO mice developed from a unique population of smooth-muscle-like cells derived from a proglucagon-expressing precursor. The loss of LKB1 within this subpopulation seems to be sufficient to drive tumorigenesis.

## INTRODUCTION

The tumour suppressor liver kinase B1 (LKB1), also known as serine/threonine kinase 11 (STK11), was first identified as a controller of zygote polarity in *Caenorhabditis elegans* (Kemphues et al., 1988). LKB1 phosphorylates 13 members of the AMP-activated protein kinase (AMPK) family (Lizcano et al., 2004), including polarity-regulating kinase partitioning defective-1 (Par-1) and its mammalian homologue, microtubule-affinity regulating kinase-2 (MARK2) (Marx et al., 2010). The actions of LKB1 as a tumour suppressor thus seem to be due to its role in the control of cell polarity, as well as of cell growth, metabolism and survival. LKB1 is one of two key upstream regulators of classical AMPK complexes in mammalian cells. Activation of AMPK in response to metabolic stress restrains growth factor signalling by stimulating the tuberous sclerosis protein complex (TSC1–TSC2) (Inoki et al., 2003), leading to the inhibition of mammalian target of rapamycin (mTOR), and consequently to blockage of protein and lipid synthesis (Shackelford and Shaw, 2009).

Consistent with these signalling roles, heterozygous mutation of the *LKB1* gene in humans leads to the development of Peutz-Jeghers syndrome (PJS), a premalignant disorder characterised by the appearance of pigmentation around the lips, gastrointestinal polyps and an increased risk of all cancers (Boardman et al., 1998). Gastrointestinal polyps are the main clinical feature of PJS and these can grow to large sizes, leading to intestinal obstruction, intussusception, infarction and bleeding. A deeper understanding of how LKB1 restricts tumour formation, and the identification of the intestinal cell types most prone to transformation, are thus needed to allow the development of novel treatments for PJS, a disease for which there are presently no approved pharmaceutical strategies.

Homozygous models of *LKB1* deletion are difficult to study because constitutive *Lkb1*^−/−^ mice are not viable beyond embryonic day 10 (Jishage et al., 2002; Ylikorkala et al., 2001). In contrast, heterozygous deletion of a single *Lkb1* allele leads to the appearance of PJS-like polyps after 5 months in mice (Bardeesy et al., 2002; Miyoshi et al., 2002). These polyps develop primarily at the gastro-duodenal junction and have similar characteristics to polyps found in PJS in humans (Miyoshi et al., 2002). However, the cellular provenance of the intestinal polyps in this model has not been established definitively. Previous studies addressing this issue showed that mono- or biallelic deletion of *Lkb1* from smooth muscle, using a conditional *Lkb1* allele and recombination mediated by *SM-CreERT2(ki)*, led to smaller polyps (~0.6 mm) than those seen in *Lbk1*^+/−^ mice (1.5–10 mm), and not to large occluding polyps or to intestinal obstruction (Katajisto et al., 2008). By contrast, no polyps were observed after targeted gastrointestinal epithelial *Lkb1* deletion using a *Cyp1a1*-specific inducible Cre recombinase (Shorning et al., 2009), suggesting that epithelial cells could play a minor role in polyp development in PJS.

Enteroendocrine cells represent a small but significant population of cells within the gut. These cells make up less than 1% of all those within the intestine but are essential for gut physiology and, collectively, constitute the largest endocrine organ in the body. Through the expression of proglucagon and prohormone convertase (PC)1/3, enteroendocrine L cells (and to a lesser extent K cells) synthesise and release hormones, including glucagon-like peptide-1 (GLP-1), GLP-2 and oxyntomodulin (OXM) (Habib et al., 2012). These hormones target receptors on pancreatic β-cells and in the central nervous system, thus regulating growth and metabolism (Diakogiannaki et al., 2012). Hormones released by enteroendocrine cells, including peptide tyrosine tyrosine (PYY), GLP-1, GLP-2 and OXM, are of considerable therapeutic interest due to their favourable influence on appetite, energy expenditure and glucose tolerance. GLP-1 is an incretin hormone, a hormone that stimulates the release of insulin from the pancreas. Thus, GLP-1 receptor agonists and inhibitors of GLP-1 hydrolysis are now front-line therapies for type 2 diabetes (T2D) (Campbell and Drucker, 2013; Nauck, 2011), and PYY and OXM analogues, which regulate appetite and control energy expenditure, are both currently being developed to treat obesity. Moreover, enhanced release of these endogenous hormones is strongly implicated in the beneficial effects of bariatric surgery on diabetes mellitus (Karra et al., 2010). Finally, GLP-2 is a peptide hormone that is important in the turnover of epithelial cells (le Roux et al., 2010).

**TRANSLATIONAL IMPACT**

**Clinical issue**

Peutz-Jeghers syndrome (PJS) is a pre-malignant syndrome that poses a considerable burden on health owing to the formation of gastrointestinal polyposis. This disease leads to premature mortality as a result of increased malignancy in all organs. Although mutations of the liver kinase B1 (*LKB1*) tumour suppressor gene have been characterised in PJS, the disease process towards malignancy is poorly understood. This makes early screening for cancer extremely difficult and currently there is no pharmaceutical treatment available for individuals with PJS. LKB1 influences several pathways that control cell growth, including AMP-activated protein kinase (AMPK) and mammalian target of rapamycin (mTOR) signalling. Understanding the role of LKB1 within different types of gastrointestinal cells is therefore of crucial importance for understanding the disease process and the pathways leading to malignancy. Previous research has studied the impact of LKB1 within the smooth muscle and the epithelium of the gastrointestinal tract. Enteroendocrine cells are specialised cells within the gut that allow the gut to communicate with the brain and the pancreas. Here, the authors studied the impact of deleting LKB1 within these cells to elucidate their role in PJS.

**Results**

In this study, the authors used mice in which LKB1 was knocked out by deleting the floxed *Lkb1* alleles using Cre recombinase under the control of the proglucagon promoter. Deletion of LKB1 in proglucagon-expressing enteroendocrine cells led to the formation of large gastro-duodenal polyps and premature mortality. These polyps had the appearance of PJS-like polyps, with an arborising smooth-muscle core. Proglucagon-expressing enteroendocrine cells were rare within the polyps. However, lineage tracing revealed that the connective tissue within the polyps was derived from proglucagon-expressing precursor cells, whereas villus-like cells were not. Lineage tracing in wild-type mice demonstrated that small numbers of proglucagon-expressing cells undergo epithelial-mesenchymal transition to become smooth-muscle-like cells within the first 10 days of life.

**Implications and future directions**

These results suggest that LKB1 plays a role in the dysregulation of proglucagon-expressing enteroendocrine precursors towards tumorigenesis. Enteroendocrine cells are a minor cell population, making up less than 1% of the cellular content within the gut. However, deletion of LKB1 within these cells is sufficient to induce polyp formation, demonstrating their crucial importance in the development of PJS. LKB1 is an important determiner of gut cell fate, and targeting LKB1 or its downstream pathways could lead to the development of novel treatments for individuals with PJS. This work suggests that further studies are warranted in humans to assess the role of the enteroendocrine system in the pathogenesis of this disease.

Up to now, the role of LKB1 specifically in enteroendocrine cells has not been examined. We (Sun et al., 2010) and others (Fu et al., 2009; Granot et al., 2009) have previously shown that LKB1 is a powerful restrictor of pancreatic β-cell growth and development, and plays a role in the control of insulin secretion. We have also noted (Leclerc et al., 2011) that both LKB1 and AMPK are involved in the control of glucagon secretion. However, and in marked contrast to the effect of LKB1 deletion in β-cells, deletion in α-cells exerts little effect on α-cell proliferation or mass (G.S. and G.A.R., unpublished observation).

To elucidate the role of LKB1 in proglucagon-expressing enteroendocrine cells, and the possible contribution of these cells or their progenitors in the formation of polyps in PJS, we have crossed mice bearing floxed *Lkb1* alleles with mice bearing a proglucagon-specific Cre recombinase. This has allowed us to determine the consequences of the loss of *Lkb1* gene function in cells expressing proglucagon, which include the enteroendocrine L cells (plus a subset of K cells), pancreatic α-cells, and GLP-1 neurons in the brainstem [notably the nucleus tractus solitarius (NTS)] and elsewhere in the central nervous system (Llewellyn-Smith et al., 2013).

We show that Cre-recombinase-mediated deletion of *Lkb1* from the enteroendocrine population causes the growth of large gastro-duodenal polyps that eventually lead to death, most likely owing to intestinal obstruction. Lineage tracing suggests that transiently expressing proglucagon enteroendocrine precursor cells undergo epithelial-mesenchymal transition (EMT) to become smooth-muscle-like cells. Loss of LKB1 within these cells leads to dysregulated growth and tumorigenesis. The present study thus contributes towards increased understanding of the mechanism underlying the PJS phenotype.

## RESULTS

### Generation of GluLKB1KO mice

In order to generate GluLKB1KO mice deleted for both alleles of *Lkb1* in proglucagon-expressing enteroendocrine cells, in pancreatic α-cells and in GLP-1^+^ neurons within the brainstem and elsewhere, we crossed *Lkb1* floxed mice with animals carrying a Cre-recombinase transgene under the control of the proglucagon promoter located within a bacterial artificial chromosome (BAC) (Parker et al., 2012).

### Deletion of LKB1 from proglucagon-expressing cells leads to premature mortality

**Fig. 1.**
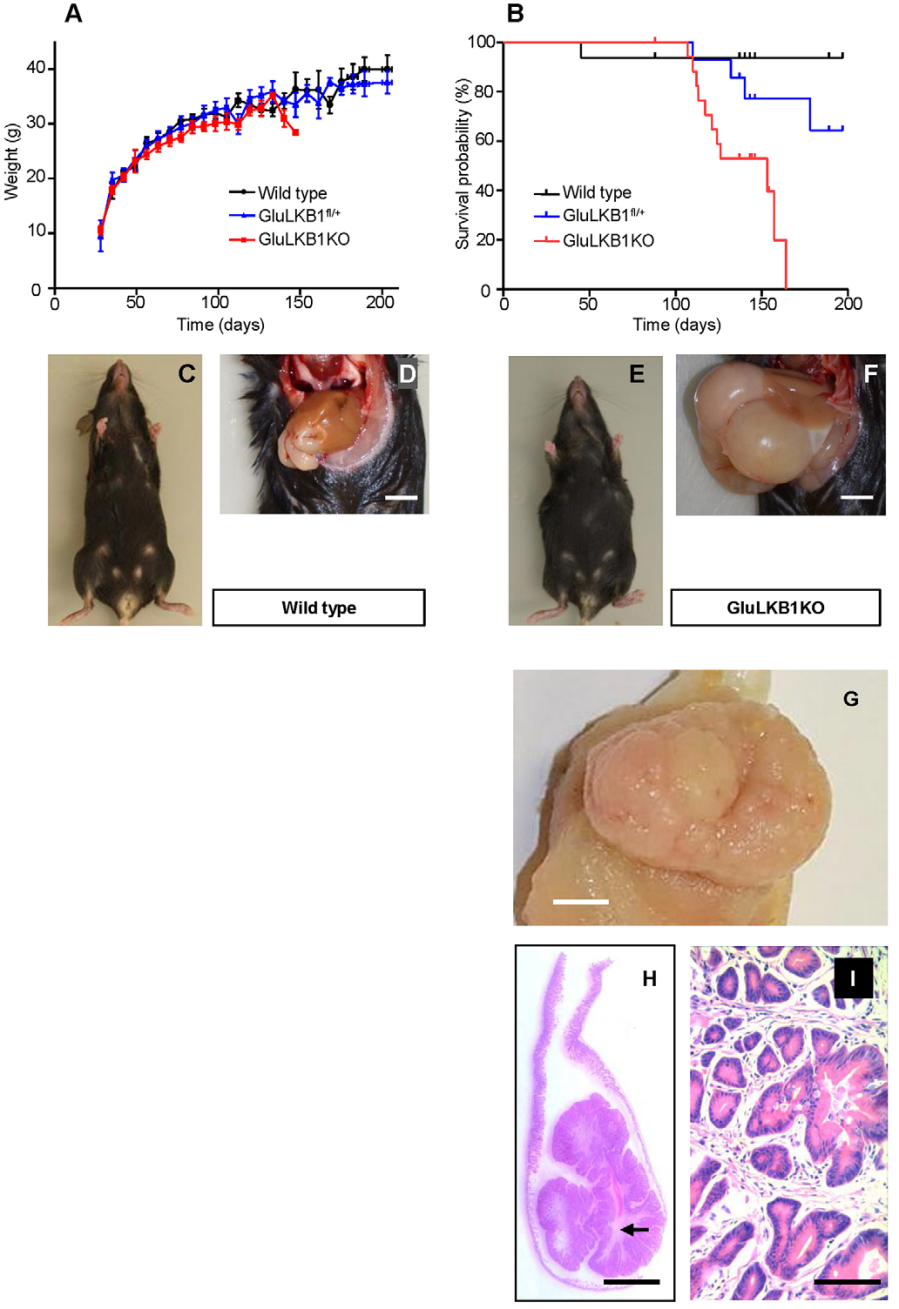
**Decreased lifespan and development of gastro-duodenal polyps following *Glu-Cre*-dependent deletion of *Lkb1*.** (A) Body weight changes in wild-type (*n*=10), GluLKB1^fl/+^ (*n*=11) and GluLKB1KO (*n*=13) mice. Analysis by one-way ANOVA did not demonstrate any significant difference between the three groups. (B) Kaplan-Meier survival curves for wild-type, GluLKB1^fl/+^ and GluLKB1KO mice. Vertical deflections on the graph represent censored data where the exact date of death was unknown owing to euthanasia of mice with ill health. GluLKB1KO mice (*n*=21; red), displayed significantly reduced lifespan and 100% of the cohort were deceased by day 164 (Log-rank Mantel-Cox test, *P*<0.001). GluLKB1^fl/+^ mice (blue; *n*=16) displayed a trend towards reduced longevity compared with the wild-type mice (black; *n*=16). By day 197, 93.7% of the wild-type mice had survived compared with 64.3% of the heterozygous mice and 0% of the homozygous knockout mice. Median survival for the GluLKB1KO mice was 153 days. (C) Representative wild-type mouse with normal phenotype. (D) Abdomen of a wild-type mouse displaying normal gastrointestinal contents. Scale bar: 5 mm. (E) Photograph of a representative GluLKB1KO mouse demonstrating a bloated appearance. (F) Photograph of the abdomen of a GluLKB1KO mouse displaying grossly distended stomach and duodenum due to the presence of a large gastro-duodenal polyp. Scale bar: 5 mm. (G) Gross macroscopy of a representative sessile polyp. (H) Haematoxylin-eosin (H&E)-stained section of a pedunculated polyp, demonstrating tubulovillous architecture with finger-like glands arising from the stalk and a core of arborizing smooth muscle (arrow). (I) Higher magnification of panel H. H&E staining with 10× magnification of the polyp. Scale bars: (G,H) 5 mm; (I) 100 μm.

GluLKB1KO mice developed normally and appeared similar to their heterozygous and wild-type littermates. Comparison of the growth curves of GluLKB1KO, heterozygous (GluLKB1^fl/+^) and wild-type mice revealed a trend towards lower body weights in the GluLKB1KO group, although this difference did not reach significance (as analysed by one-way ANOVA; Fig. 1A). Most of the conditional null animals appeared in good health and were well groomed. However, from around 120 days after birth, GluLKB1KO mice became bloated in appearance (Fig. 1E) and premature mortality was observed (Fig. 1B). Thus, 11/21 (52%) of the GluLKB1KO mice had died unexpectedly by day 164. The remainder (10/21) displayed symptoms of distress (bloating, weight loss or reduced activity) and were euthanized upon veterinary advice and subjected to post-mortem examination. By contrast, 1/16 (6.2%) wild-type mice died by 164 days of age. Heterozygous animals displayed an intermediate mortality, with 4/16 (25%) dying before day 200 (Fig. 1B).

### GluLKB1KO mice develop large polyps at the gastro-duodenal junction

Pathological analysis of the gastrointestinal tract during post-mortem revealed that 8/10 homozygous GluLKB1KO mice examined developed gastro-duodenal polyps from around 120 days (107–164 days). These were not apparent earlier in life nor did they seem to cause any signs of obstruction or symptoms of distress until a few days prior to death, where weight loss was observed in some cases (6/13 animals; Fig. 1A). As a result, tumours were usually discovered on gross pathological analysis of the gastrointestinal tract during post-mortem (Fig. 1E,F), and were absent from wild-type animals (Fig. 1C,D). In each case, the polyps were large [mean (±s.d.) length 15.8±7.6 mm; mean diameter 10.6±2.0 mm; *n*=5]. Polyps were either pedunculated (Fig. 1H) or sessile (Fig. 1G). The polyps were also hyperplastic and complex in structure (Fig. 1G,H,I). It is therefore likely that small-bowel obstruction caused by the polyps led to the weight loss and demise of GluLKB1KO animals. In contrast, we identified tumours in only one GluLKB1^fl/+^ mouse out of 16 examined. Detailed post-mortem analysis of elderly (471 day, *n*=3) GluLKB1^fl/+^ mice revealed that these mice had normal gastrointestinal tracts and no evident signs of polyp formation. In two of the GluLKB1KO mice examined, hypersplenism was evident. This feature has been previously described in *Lkb1*^+/−^ mice following haemorrhage from enlarged polyps (Jishage et al., 2002).

A formal histopathological analysis of the tumour and of vital organs from a representative GluLKB1KO mouse revealed that polyps formed at the junction of the forestomach and glandular stomach and extended into the duodenum over the Brunner’s glands (Fig. 1G). Areas of stromal overgrowth, consisting of complex branching tubuloglandular components, were apparent (Fig. 1H,I). Polyps were predominantly composed of foveolar elements rather than specialised glands and were chiefly found in the pyloric area. Large numbers of apoptotic/senescent/oncotic cells were also seen in areas, creating a vacuolated appearance (not shown). The polyp exhibited a ‘hyper-mature’ architecture with deeper glandular components being relatively less affected. In areas of stromal overgrowth the morphology was similar to that of the human PJS type polyps (Beggs et al., 2010). In addition, occasional small (1- to 2-mm diameter) polypoid formations were noted in the large intestine. There were also complex formations and dilated lymphatics in the lamina propria component (not shown) but no definite dysplasia within any of the lesions. This is similar to the findings in individuals with PJS, where polyps rarely exhibit dysplasia (Bardeesy et al., 2002). Histopathological analysis of the brain, pancreas, liver, lungs, heart, kidneys and testes was unremarkable.

In order to confirm deletion of LKB1 in the expected target tissues, we performed RT-PCR analysis on mRNA extracted from GluLKB1KO mice. This analysis revealed the predominant expression of the shorter form, derived from the recombined gene, in the tumours from GluLKB1KO mice (supplementary material Fig. S1). Recombination was also detected, albeit to a lesser extent, in the NTS within the brainstem and also the olfactory bulb (not shown), reflecting the relative scarcity of proglucagon-expressing cells in these regions (supplementary material Fig. S1). The shorter PCR product was also detected as a relatively minor fragment in the colon and, to a much larger extent, in the duodenum, thus suggesting extensive recombination in the latter region of the intestine (supplementary material Fig. S1). The shorter fragment was not detected in any of the above tissues from wild-type mice (not shown).

### GluLKB1KO mice display normal fasting plasma GLP-1 levels and oral glucose tolerance

**Fig. 2.**
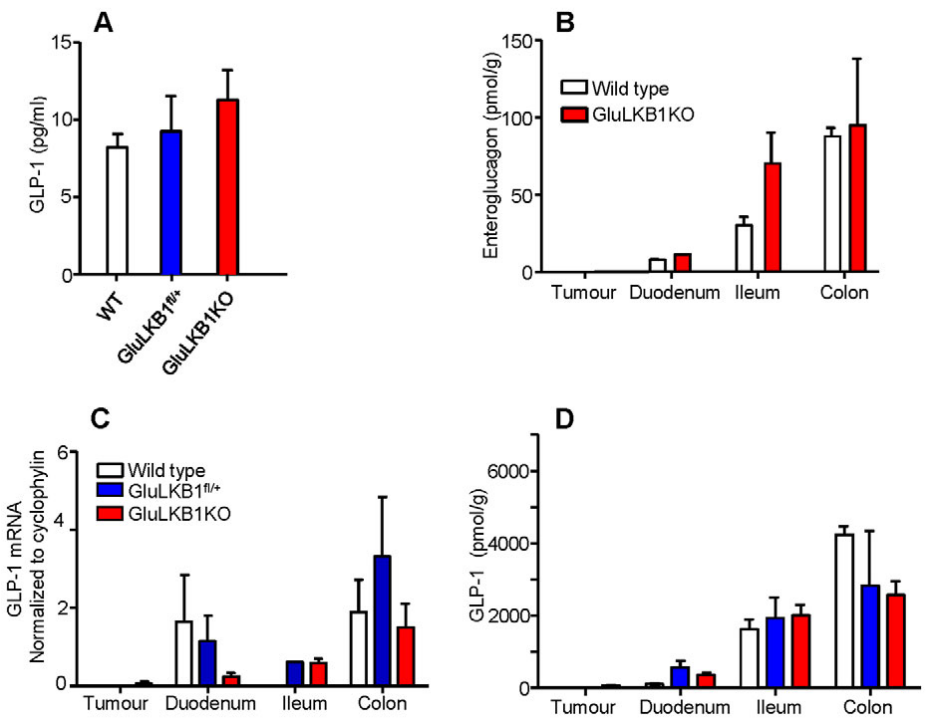
**Effects of *Glu-Cre*-mediated LKB1 deletion on intestinal peptide hormone expression.** (A) Fasting plasma GLP-1 levels of wild-type, GluLKB1^fl/+^ and GluLKB1KO mice. (B) Enteroglucagon levels measured by RIA following acetic-acid extraction of gastrointestinal tissue of wild-type and GluLKB1KO mice. (C) Quantitation of proglucagon (GLP-1) by qRT-PCR for wild-type, GluLKB1^fl/+^ and GluLKB1KO mice. (D) GLP-1 levels measured by RIA following acetic-acid extraction of gastrointestinal tissue. Mice were aged between 125 and 183 days. Two-way ANOVA did not detect any significant differences between the groups.

We next determined whether GLP-1 can be stored and released from the tumours (or from L cells in the rest of the gut), and might thus affect glucose homeostasis. However, arguing against substantially altered incretin release, GluLKB1KO mice developed unremarkably up to ~120 days of age, maintaining normal growth (see above, Fig. 1A) and metabolic parameters were only mildly affected compared with wild-type animals. GluLKB1KO mice showed no difference in fasting glucose levels compared with controls and, whereas intraperitoneal glucose tolerance was slightly improved in male GluLKB1KO at 5 weeks of age, the reverse was true in female GluLKB1KO mice versus controls (supplementary material Fig. S2A,B,D,E). No difference in oral glucose tolerance was noted between genotypes (supplementary material Fig. S2C,F); therefore, incretin release is also likely to have remained unchanged. Likewise, fasting plasma GLP-1 levels were also normal in GluLKB1KO mice (Fig. 2A).

In support of the unchanged glucose tolerance of GluLKB1KO mice compared with control animals, GLP-1 mRNA and protein levels were not significantly altered in the intestines of the GluLKB1KO mice compared with the wild-type controls (Fig. 2C,D). However, there was significant variance in these parameters between mice, which might have led to a type-2 statistical error. To determine whether altered levels of unprocessed GLP-1 precursors were present in the GluLKB1KO mice compared with controls, samples were assayed for enteroglucagon (Bryant et al., 1983) (Fig. 2B). Arguing against this possibility, intestinal enteroglucagon levels were not significantly altered in GluLKB1KO mice, although again there was considerable variation between animals. In the tumours, GLP-1 mRNA and protein levels were extremely low and were similar to those measured in wild-type duodenum (Fig. 2C,D).

### Lineage tracing suggests that polyps are derived from cells transiently expressing proglucagon that have undergone EMT

**Fig. 3.**
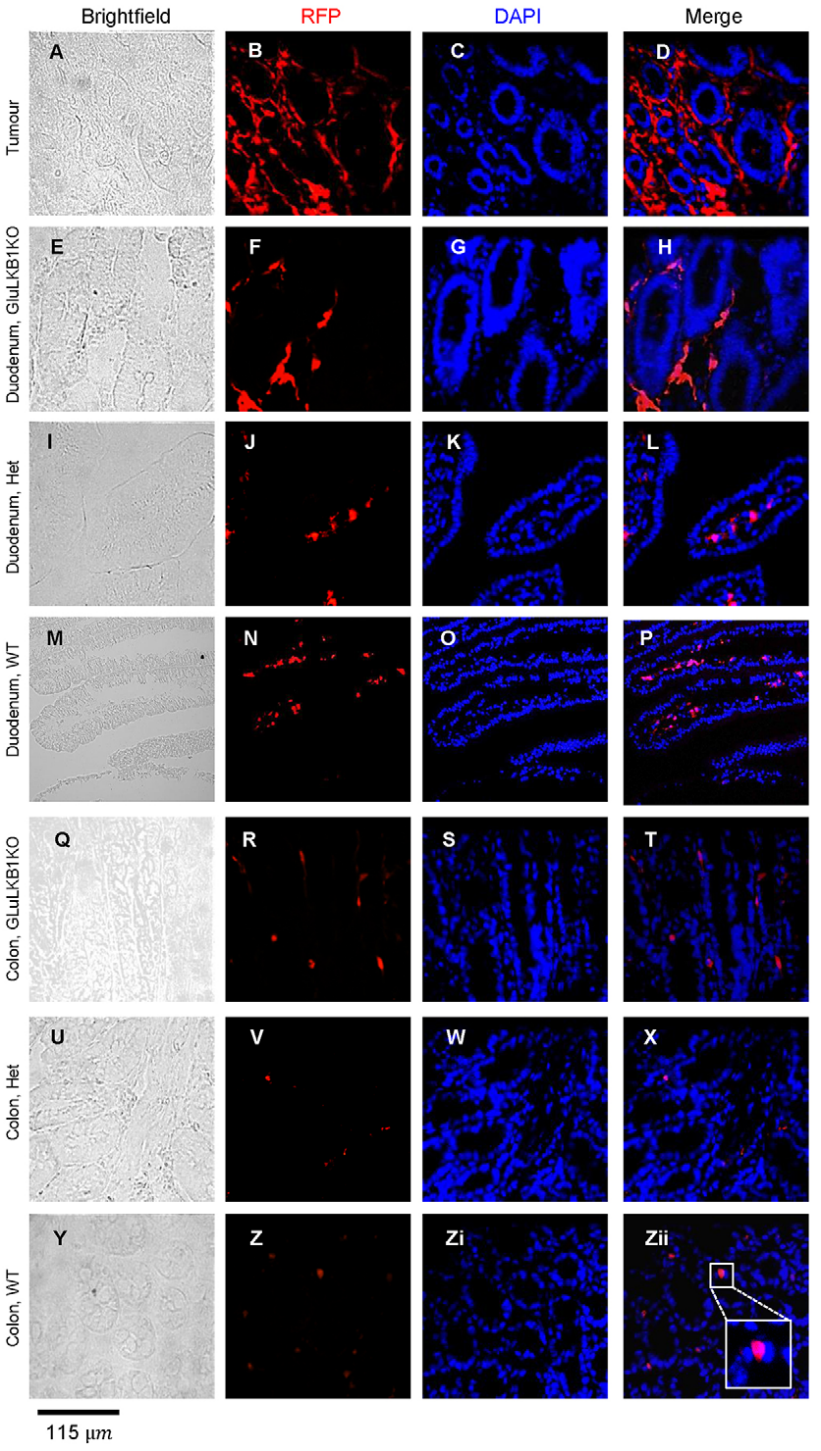
**Fate of proglucagon-gene-expressing intestinal cells explored by lineage tracing.** Immunohistochemistry of frozen sections using an anti-RFP antibody and confocal microscopy images of polyp, duodenum and colon from wild-type (‘WT’; M-P,Y-Zii), GluLKB1^fl/+^ (I-L,U-X) and GluLKB1KO (A-H,Q-T) mice. Brightfield, RFP, DAPI (nuclei localisation) and merged RFP/DAPI images are shown. In Zii, inset shows an enlarged image of an RFP-stained cell located within a crypt and with predicted enterocyte morphology.

To further investigate the mechanisms through which deletion of LKB1 in proglucagon-expressing cells could lead to the generation of duodenal polyps, we performed lineage-tracing studies using Rosa26tdRFP reporter mice. In these mice, Cre-mediated recombination leads to the excision of a STOP codon and hence the expression of RFP (Luche et al., 2007). The presence of RFP in the polyps of GluLKB1KO:Rosa26tdRFP mice confirmed that a substantial proportion of tumour cells were derived from proglucagon-expressing cells, as expected. However, unexpectedly, the majority of RFP-labelled cells in the polyps were located in the connective-tissue areas surrounding the villi. These cells displayed an elongated spindle-shaped structure more similar to smooth muscle than enteroendocrine cells (Fig. 3A–D). It is possible that these RFP-mesenchymal cells recruit epithelial cells of non-RFP lineage that then populate the tissue. Occasional RFP-positive cells were apparent in the villi and were likely to correspond to enteroendocrine cells (e.g. see wild-type colon, Fig. 3Zii).

**Fig. 4.**
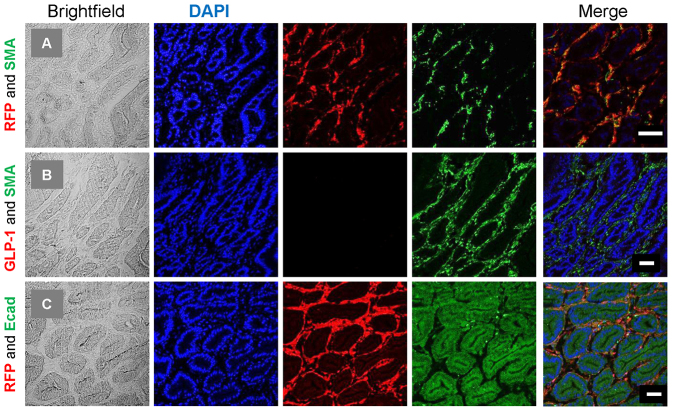
**Immunohistological characterisation of RFP cells within polyps.** Colocalisation experiments were performed on 7-μm frozen sections. (A) Anti-RFP (rabbit polyclonal) and SMA (mouse monoclonal), (B) GLP-1 (goat polyclonal) and SMA (rabbit polyclonal), and (C) RFP (rabbit polyclonal) and E-cadherin (mouse monoclonal). Brightfield, DAPI, red and green fluorescence, and the merged images are shown. RFP cells were found to colocalise with SMA; however, the SMA-labelled cells did not colocalise with either GLP-1 or E-cadherin. Scale bars: 50 μm.

Furthermore, analysis of the duodenum (Fig. 3E–H) of GluLKB1KO mice revealed a similar picture, with expression of RFP in connective-tissue cells alongside cells within the villi/crypts that are likely to correspond to enteroendocrine cells. The duodenum of GluLKB1^fl/+^ mice contained RFP-stained cells in the centre of the villi, but not surrounding them; these cells are likely to correspond to smooth-muscle cells (Fig. 4I–L). RFP-labelled cells were also present in the duodenum and colon of wild-type mice (expressing RFP under the *Cre* glucagon promoter but with wild-type *Lkb1* alleles; Fig. 4M–P and 4Y–Zii). In these wild-type mice, RFP-labelled cells were seen in populations of cells that are likely to correspond to enteroendocrine cells, as well as in those of mesenchymal origin. However, numbers of these mesenchymal cells were fewer than seen in the KO mice. This is described more fully in the following section. Wild-type mice lacking RFP displayed no fluorescence (not shown), confirming specificity of the primary antibody.

### Characterisation of RFP-positive cells within the polyp

To further characterise the RFP-positive cells within the polyps, a series of colocalisation experiments was performed. RFP cells colocalised with α-smooth muscle actin (α-SMA), in keeping with their spindle-shaped mesenchymal appearance (Fig. 4A). In contrast, and despite the fact that these cells were of a proglucagon-expressing lineage, the SMA cells did not colocalise with GLP-1 (Fig. 4B), which was largely absent from the polyps. In keeping with this, and as previously discussed, peptide measurements [radioimmunoassay (RIA)] and mRNA of proglucagon within the tumour were low (Fig. 2C,D). Although occasional GLP-1-containing cells were seen within the tumour, these were rare. E-cadherin, an epithelial cell-adhesion receptor, is thought to be essential to maintain the integrity of the epithelium. It has been previously shown that its repression is required for cells to undergo EMT. Within the polyps, E-cadherin was found to stain enterocytes but did not colocalise with RFP cells (Fig. 4C).

### Assessment of *Glu-Cre* fidelity

**Fig. 5.**
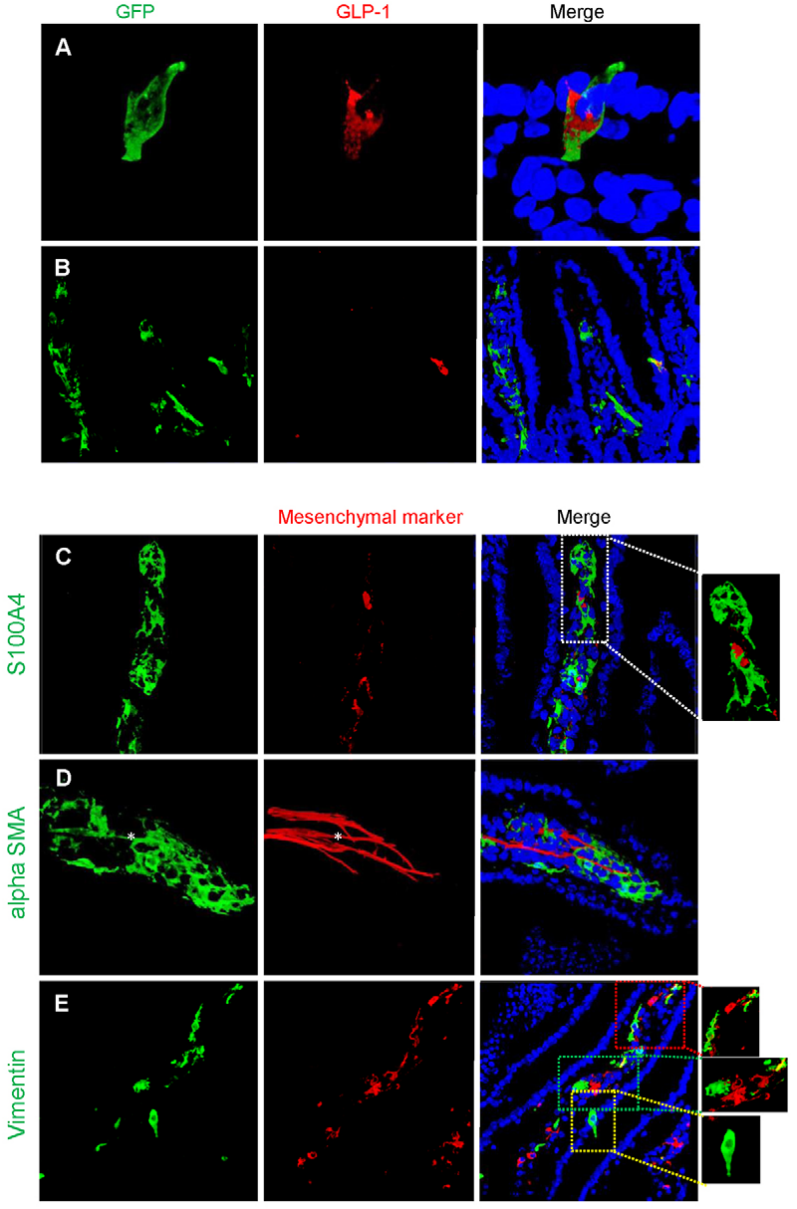
**Expression of fluorescence in adult Glu-Cre mouse small intestine.** (A) Photomicrographs demonstrating green fluorescence in a typical epithelial cell, resembling an enteroendocrine cell, and co-staining for proglucagon (GLP-1). The sequence in the panel shows the green fluorescence, proglucagon stained with a secondary Alexa-Fluor-555 and finally the merged image with Hoechst staining of the nuclei. (B) In certain cells, fluorescence is also demonstrated in mesenchymal cells within the villi; these cells do not co-stain for proglucagon. (C–E) Further characterisation of these GFP-positive but GLP-1-negative cells demonstrates that they do not co-stain with the fibroblast-specific marker S100A1 (C), but a proportion of cells co-stain with α-SMA (D) and others with vimentin (E). In this final panel (E), two areas of GFP and vimentin colocalisation are highlighted (red and green boxes) and a typical enteroendocrine cell is highlighted (yellow box). The asterisk in D indicates a GFP-positive cell representing a lacteal and co-staining with α-SMA.

To assess reliability of the proglucagon promoter and to ensure that random expression of *Cre* was not occurring, animals carrying *Glu-Cre*, driving expression of a green fluorescent reporter protein (GFP), were examined. As expected, most proglucagon-containing cells exhibited green fluorescence (Fig. 5A). However, and unexpectedly, a proportion of GFP-positive cells within the villi did not stain for proglucagon and had a mesenchymal appearance (Fig. 5B). To further characterise these fluorescent non-proglucagon-expressing cells, cells were co-stained for the fibroblast marker S100A4 (fibroblast-specific protein-1) (Fig. 5C), SMA (Fig. 5D) and a general mesenchymal marker found in myofibroblasts, vimentin (Fig. 5E). The GFP-positive cells did not stain for S100A4 but some GFP-positive cells resembling lacteals stained for SMA, and others for vimentin.

**Fig. 6.**
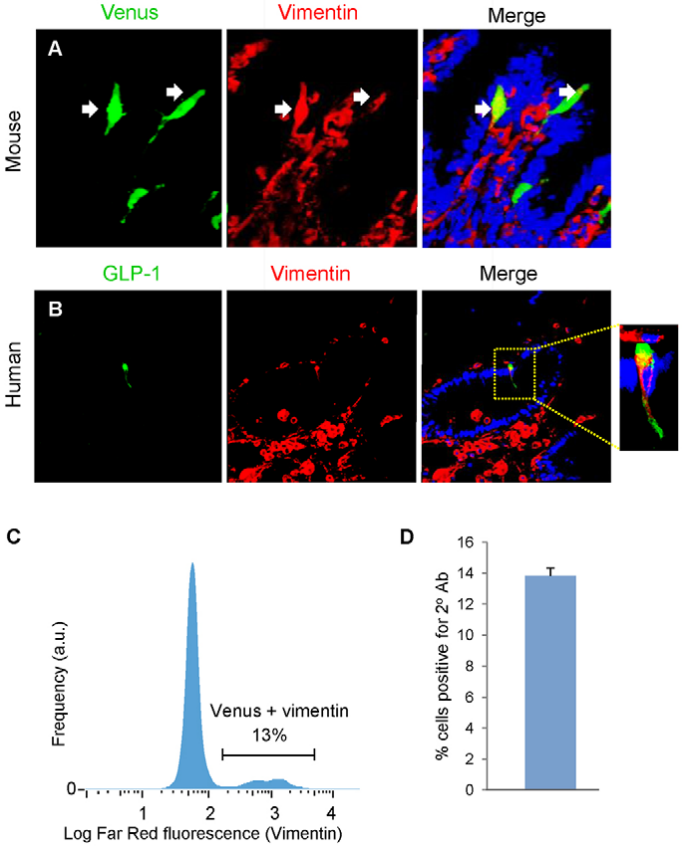
**Characterisation of Venus proglucagon-expressing enteroendocrine cells.** Adult mGLU124 upper small intestine was stained for GFP and vimentin, and a green and red secondary. (A) Some sporadic Venus cells colocalised with vimentin. Upper SI suspensions from mGLU124 mice were stained with an antibody against vimentin and a far red secondary, as analysed by FACS. Arrows indicate representative enteroendocrine L cells. (C) Histogram of the far red fluorescence (vimentin) of gated Venus cells. (D) Mean and s.e.m. of Venus cells colocalising vimentin from FACS analysis of three animals. (B) Human intestine was stained for GLP-1 and vimentin, and green and red secondary antibodies. Some GLP-1-positive enteroendocrine cells colocalised with vimentin.

To further examine L cells, and the possibility that these can undergo EMT, an alternative model was used. mGLU124 mice express Venus (a yellow fluorescent protein) driven by the proglucagon promoter (Reimann et al., 2008). These cells only fluoresce when proglucagon is expressed. In these mice, fluorescence was only seen in the enteroendocrine L cells and not at all in the mesenchyme layer. Subpopulations of L cells were found to express vimentin, a traditional mesenchymal marker and inducer of EMT (Mendez et al., 2010) (Fig. 6A). FACS analysis of mouse small intestines (top 10 cm) revealed that, on average, 13% of Venus cells expressed vimentin (Fig. 6C,D). This is a small percentage of all vimentin-expressing cells (0.39%). This finding was replicated using human tissue samples (Fig. 6B).

**Fig. 7.**
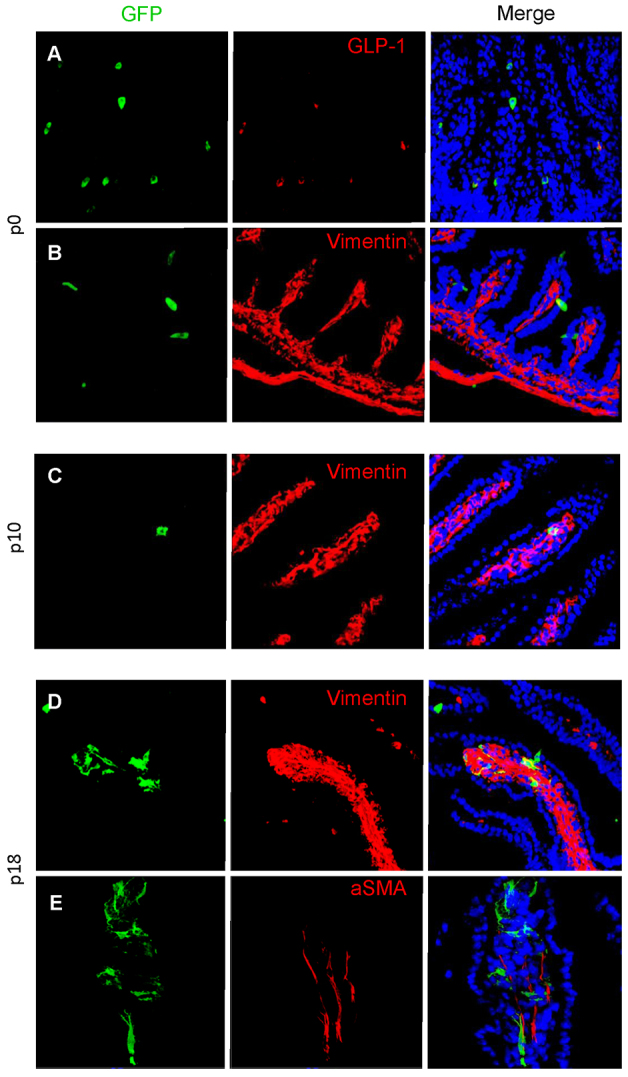
**Evidence for epithelial-to-mesenchymal transition.** At P0, GLU-Cre/ROSA26-GCaMP3 mice have GFP expression limited to epithelial cells within the small intestine, shown by cells colocalising with GLP-1 (A). No mesenchymal cells were observed within the mesenchymal core of villi (*n*=3 animals), although some epithelial GFP cells colocalise with the mesenchymal marker vimentin (B). In P10 mice, GFP cells are present that do not colocalise with proglucagon but have a mesenchymal appearance and co-stain with the mesenchymal marker vimentin (C). Finally, in P18 mice, larger numbers of GFP-expressing mesenchymal cells are present. A subset of these co-stain with vimentin (D), whereas others co-stain with SMA (E).

To study the possibility that vimentin-expressing L cells are able to transform into mesenchymal cells, we used an alternative GFP reporter model using the same deleter strain, GluCreROSA26-GCaMP3 mice (see Materials and Methods), at various stages of development. In three P0 (newborn) mice, GFP expression was limited to epithelial cells and colocalised with proglucagon (Fig. 7A). Some of these GFP-proglucagon cells also expressed an EMT marker, vimentin (Fig. 7B). By day 10 (P10) small numbers of GFP-positive mesenchymal cells were present within a minority of villi (Fig. 7C). The majority of these co-stained with vimentin but not with proglucagon. By day 18 (P18), GFP cells were observed in villi, often clustered together (Fig. 7D). These were more differentiated, resembled lacteals, and some co-stained with SMA but not S100A4 (Fig. 7E). Thus, small numbers of proglucagon-expressing cells undergo EMT within the first 10 days of life.

## DISCUSSION

### Deletion of LKB1 from proglucagon-expressing cells leads to the development of large gastro-duodenal polyps

The principal aim of the present study was to determine the role, if any, of LKB1 in proglucagon-expressing cells. Using a series of lineage-tracing approaches, we show that *Cre* expression is likely to mark, in addition to L cells, a population of cells that are derived from proglucagon-expressing precursors. Importantly, similar behaviour was observed in three independent Glu-Cre founder lines and is unlikely, therefore, to reflect mis-expression resulting from random insertion events.

Although little evidence was obtained of a role for LKB1 in the development of these cells in the brain, and only a minor role, if any, in the pancreas, we demonstrate that loss of LKB1 is critically important in the regulation of intestinal proglucagon-expressing enteroendocrine cells (and/or proglucagon-expressing progenitor cells). Thus, homozygous *Lkb1* deletion led to the development of large polyps at the gastro-duodenal junction; these polyps shared similarities with both PJS polyps in humans and with those previously described in mice lacking a single allele of *Lkb1* throughout the body (Miyoshi et al., 2002).

Important quantitative differences were observed between the growth of polyps in GluLKB1KO mice and earlier models of *Lkb1* deletion in the intestine (see Introduction and below). Interestingly, although enteroendocrine cells represent less than 1% of the total cellular gastrointestinal tract, *Lkb1* deletion in the proglucagon-expressing cell population led to the development of tumours averaging in size between 1 to 3 cm in mice aged between 120 and 156 days, selectively in the duodenum. These tumours were of a similar size to those reported in 6-month-old systemic *Lkb1*^+/−^ mice (2- to 3-cm diameter) (Rossi et al., 2002). However, they were considerably larger than those previously described in smooth-muscle-targeted LKB1-knockout mice (0.6 mm) after deletion of either one or both conditional alleles (Katajisto et al., 2008). Furthermore, deletion of LKB1 within intestinal epithelial cells has not been described to lead to polyposis (Shorning et al., 2009). Interestingly, in this model, deletion of LKB1 throughout the intestinal epithelium has previously been shown to inhibit the terminal differentiation of secretory goblet cells, leading to a more immature, less differentiated cell phenotype, suggesting that LKB1 plays a role in EMT in these cells (Shorning et al., 2009).

Thus, polyps derived from proglucagon-expressing cells or progenitors show a remarkably enhanced potential for growth and survival compared with existing models. However, polyps were rarely encountered in heterozygous GluLKB1^fl/+^ mice (1/16), despite accelerated mortality of these mice compared with wild-type animals. The latter result might seem to question the quantitative contribution of proglucagon-expressing-cell-derived tumour cells in human PJS, in which *LKB1* loss of heterozygosity (LOH) has been found not to be a required event for polyp formation (Katajisto et al., 2008). Nonetheless, and despite the absence of evident polyposis, GluLKB1^fl/+^ mice also displayed a tendency towards a shortening of lifespan (Fig. 1B).

### Loss of LKB1 in proglucagon-expressing cells does not alter glucose tolerance

In the pancreatic β-cell, LKB1 deletion leads to enhanced insulin secretion and improved glucose tolerance (Fu et al., 2009). Other studies (G.S. and G.A.R., unpublished observations) have revealed that deletion of *Lkb1* selectively in the pancreatic α-cell (using a shorter and essentially pancreatic α-cell-restricted proglucagon promoter) (Herrera, 2000) exerts only minor effects on glucagon release and blood glucose levels, consistent with the mild perturbations in the intraperitoneal glucose tolerance seen here (supplementary material Fig. S2A,D). GLP-1 was virtually undetectable in the polyps, and plasma GLP-1 levels were similar in knockout mice compared with controls. In accordance with this, body weight and oral glucose tolerance were similar in knockout mice compared with controls. Had the levels of incretins been increased in the knockout mice, we would have expected certain metabolic consequences, such as reduced body weight and improved glucose tolerance (Suzuki et al., 2012).

Given the importance of LKB1 in neuron development and polarisation (Barnes et al., 2007; Sun et al., 2011), the absence of any evident neuronal phenotype in the present model was surprising. We noted that there was no apparent change in the number or morphology of GLP-1^+^ neurons in the NTS (supplementary material Fig. S3), despite recombination in this nucleus. However, the premature mortality of the null mice is likely to have masked any changes, such as changes in bodyweight, secondary to subtle alterations in the function of GLP-1^+^ neurons, or in glucagon secretion (G.S. and G.A.R., unpublished). Of note, we also observed recombination within the olfactory bulb (not shown), where more GLP-1^+^ neurons are located (Trapp and Richards, 2013; Merchenthaler et al., 1999), although whether this led to any change in olfaction has not been investigated.

### Predilection for polyps at the gastro-duodenal junction

Deletion of LKB1 in cells of the proglucagon-expressing lineage led to the generation of large polyps chiefly in the duodenum. This was a surprising finding because numbers of proglucagon-expressing cells in this part of the gut are not excessive. Proglucagon-expressing cells are distributed throughout the gastrointestinal tract with increasing numbers distally and with the highest concentration within the colon and rectum (Eissele et al., 1992). Proglucagon-expressing enteroendocrine-cell density is genetically determined and region-specific (Hansen et al., 2013). We have also considered the possibility that an increased density of cells at the gastro-duodenal junction could occur after LKB1 deletion because of a disruption in the polarity of these cells, leading to their hyperproliferation in this part of the gut. However, no evidence for this hypothesis was obtained. The present studies in wild-type mice using RFP as a lineage tracer (Fig. 4) provided little evidence for the presence of large numbers of proglucagon-expressing cells in the duodenum.

In *Lkb1*^+/−^ mice, a similar predilection for the development of polyps at the gastro-duodenal junction has previously been described (Jishage et al., 2002). It is hypothesised that this is due to the increased mechanical forces at this point in the gut (Biancani et al., 1980). The development of polyps at this location in our GluLKB1KO model might be due to a variety of factors, including mechanical forces and the nutritional environment. It is not clear currently whether or not cells within the gastro-duodenal junction are more prone to undergo EMT.

### Epithelial-mesenchymal transition

L cells develop from common progenitor cells at the base of the intestinal crypt. Neurogenin 3, expressed late in differentiation, commits the cell towards an endocrine fate (Lee et al., 2002). Although our data do not permit a definitive assignment to be made of the progenitor cell(s) from which polyps develop after LKB1 deletion, the rapid turnover of mature L cells (4–5 days) and inability of these cells to divide (Petersen et al., 2014) would seem to make these less likely candidates as the immediate precursor. On the other hand, LKB1 deletion must occur prior to the extinction of promoter activity, i.e. while Cre is still expressed. Because we observed relatively little proglucagon staining within polyps, we suspect that the daughters of immature *Lkb1*-null L cells that have undergone transition then go on to become transformed, and develop tumours.

Our studies of Glu-Cre animals expressing GFP reporter proteins demonstrate that a small proportion of proglucagon-expressing cells express an EMT marker, vimentin, and undergo EMT by day 10. In our GluLKB1KO model, lineage-tracing studies demonstrated that loss of LKB1 from proglucagon-expressing precursors leads to increased numbers of cells within the duodenum (and more abundantly in the polyps) with mesenchymal characteristics and localisation (Fig. 4 and supplementary material Fig. S1). It is thus likely that LKB1 signalling is required to prevent proliferation of this cellular compartment. Polyps seem likely to develop from this reverted population of smooth-muscle-like cells, rather than from mature L cells, and might conceivably be influenced by tissue damage or inflammation.

### Unanswered questions

In PJS, LKB1 deficiency has been suggested to lead to the expansion of the progenitor cell compartment and disruption of stem-cell division leading to mucosal prolapse. Alternatively, loss of transforming growth factor-β (TGFβ)-mediated communication between smooth muscle cells and the epithelium has been described after conditional deletion of the former compartment (Katajisto et al., 2008). PJS polyps rarely carry oncogenic *RAS* mutations and, although individuals with PJS have increased susceptibility to malignancy, the gastrointestinal polyps themselves rarely show malignant transformation (Entius et al., 2001). Likewise, *APC* and *K-RAS*, genes frequently mutated in adenocarcinomas, are rarely mutated in PJS (Entius et al., 2001; Gruber et al., 1998). Future studies will be required to determine whether these or other mutations are involved in the development of the tumours described here in GluLKB1KO mice.

### Potential of targeting the LKB1 pathway in enteroendocrine cells in PJS and T2D

Individuals with PJS have an increased cumulative risk of cancer development. A recent meta-analysis reported that the prevalence of cancer was over 90% (Giardiello et al., 2000). It is not clear how loss of LKB1 leads to tumour development. The findings of the present study suggest an increased vulnerability of proglucagon-expressing precursors to proliferate following loss of LKB1. It is possible that this unique cell population provides a reservoir of precursors for polyp generation, thus contributing to overall tumour burden. It should be noted that testing this possibility in humans by using lineage-tracing approaches as adopted here in the mouse is not feasible. To date, few studies have considered the contribution of endocrine cells towards the PJS phenotype and the evidence available is conflicting. This might reflect the difficulties with immunohistological techniques used to detect these cells (Krstić et al., 2013; Udd et al., 2010).

It has been suggested that tumour formation in PJS is associated with the downstream effects of the loss of LKB1 and the removal of inhibition of the mTOR pathway. Rapamycin is an mTOR inhibitor used in immunosuppressive regimes. In *Lkb1*^+/−^ mice, rapamycin has been shown to reduce tumour burden (Robinson et al., 2009). There is currently insufficient evidence to support the use of rapamycin as a preventative treatment in individuals with PJS (Beggs et al., 2010). However, the development of sophisticated and targeted mTOR inhibitors could prove useful for the treatment of PJS. On the other hand, the apparent plasticity of proglucagon-expressing enteroendocrine cells (or precursors), which is reminiscent of that of pancreatic α-cells (Thorel et al., 2010), suggests that attempts to increase the number of these cells (for example in obesity or T2D), through the regulation of tumour suppressors or growth factors, should be approached with due caution.

## MATERIALS AND METHODS

### Mouse breeding strategy

Experiments were conducted according to UK Home Office regulations. Mice expressing Cre recombinase (*iCre*) under the control of the proglucagon promoter were developed as previously described (Parker et al., 2012). These were crossed with mice bearing floxed *Lkb1* alleles (Mouse Models of Human Cancer Consortium; http://mouse.ncifcrf.gov/) (Sun et al., 2011; Sun et al., 2010). The resulting GluLKB1^fl/fl^ mice were further bred against C57BL6 Rosa26tdRFP reporter mice (Luche et al., 2007). GluCreROSA26-GCaMP3 mice were generated by breeding the deleter strain with Rosa26-GCaMP3 animals (Zariwala et al., 2012). Mice expressing Venus from a BAC transgenic under the proglucagon promoter were as described (Reimann et al., 2008).

### Mouse maintenance and diet

Mice were housed in cages with 2–5 mice per cage in a pathogen-free facility with a 12-hour light and dark cycle. Animals had unrestricted access to standard mouse chow diet (Research Diet, New Brunswick, NJ). All *in vivo* procedures were conducted at the Imperial College Central Biomedical Service and were performed in accordance with the Animal Scientific Procedures Act of 1986. Mice were assessed by weekly body weights and also by the mouse body condition score (Ullman-Culleré and Foltz, 1999). Animals were weighed once or twice weekly and were euthanized if they showed signs of illness.

### Oral and intraperitoneal glucose tolerance tests

Mice were fasted for 15 hours, with free access to water, before 1 g/kg body weight glucose was administered either through oral gavage or intraperitoneally. Blood was sampled from the tail vein at 0, 15, 30, 60, 90 and 120 minutes after glucose administration (Richards et al., 2005). Blood glucose concentration was measured with a handheld glucometer (Accu-Chek; Roche, Burgess Hill, UK).

### Measurement of GLP-1

Mice were fasted for 15 hours. Blood was sampled from the tail vein into ice-cold heparinised tubes (Sarstedt, Beaumont Leys, UK) containing the dipeptidyl peptidase IV (DPP IV) inhibitor, diprotin A (Bachem, Bubendorf, Switzerland). Plasma was separated by centrifugation of blood at 2000 ***g*** for 5 minutes and total GLP-1 levels assayed using a two-site microtitre plate-based immunoassay with electrochemical luminescence detection (Meso Scale Discovery kit, Gaithersburg, MD).

### RNA extraction, RT-PCR and qRT-PCR

Total cellular RNA was extracted with TRIzol reagent (Invitrogen, Paisley, UK) and phenol-chloroform using a standard protocol. RNA was treated with DNase using a kit (Applied Biosystems, Warrington, UK). Reverse transcription was performed using a high-capacity reverse-transcriptase kit (Applied Biosystems, Warrington, UK). The reaction conditions were as follows: 25°C for 10 minutes, 37°C for 2 hours and 85°C for 5 seconds. *Lkb1* deletion was assessed using two pairs of primers within exon 1 (*Lkb1* forward 5′-AGGTGAAGGAGGTGCTGG-3′) and exon 8 (*Lkb1* reverse 5′-TCTGGGCTTGGTGGGATA-3′). The PCR conditions were as follows: 94°C for 5 minutes, then 30 cycles of 94°C for 30 seconds, 59°C for 1 minute, 72°C for 1 minute. A prolonged extension step was carried out at 72°C for 7 minutes. The expected product sizes were 796 bp for the floxed allele and 228 bp after Cre recombination, owing to the loss of exons 2–6 in the transcript (Bardeesy et al., 2002).

Expression levels of proglucagon were quantified by real-time PCR following the SYBR Green method using 25 ng of RNA. Cyclophylin was used as a reference gene. Primers were designed using Primer Express 3.0 software (ABI, Warrington, UK). The sequences for the primers used for RT-PCR are listed in supplementary material Table S1. Standard curves were constructed for each primer pair to test the efficiency of the reaction. Reaction conditions were as follows: 95°C for 2 seconds, then cycles at 95°C for 3 seconds and 60°C for 30 seconds. The melt curve conditions were 95°C for 15 seconds, 60°C for 1 minute, 95°C for 15 seconds and 60°C for 15 seconds. Fold change was measured by calculating 2^sΔΔCt^, normalising for cyclophylin and to the wild-type duodenum.

### Immunohistochemistry

Mice under anaesthesia {ketamine [60 mg kg^−1^; intramuscularly (i.m.)] and medetomidine (250 μg kg^−1^, i.m.)} were given heparin (500 IU/l), flushed with phosphate-buffered saline (PBS) to remove blood and perfused transcardially with 60 ml of phosphate-buffered 4% paraformaldehyde (PFA), pH 7.4. Tissues were retrieved and post-fixed overnight at 4°C in 4% PFA, then saturated in 20% sucrose overnight. Before cutting on a cryostat, tissues were trimmed to size, embedded in OCT, and frozen using isopentane and liquid nitrogen.

### Gut sections

Gut sections were cut at 7 μm and mounted onto Superfrost Plus glass slides. Slides were washed in PBS and then blocked for 1 hour in PBS containing 10% animal free block (Vector Laboratories, Peterborough, UK) and 0.1% Triton X-100. Slides were incubated with primary antibody. Antibodies used: RFP: Living Colors^®^ DsRed Polyclonal Antibody 632496 (Clontech, St Germain-en-Laye, France) diluted in blocking buffer (1 in 1000 overnight); rabbit polyclonal SMA Ab5695 (Abcam, Cambridge, UK) (1 in 500), mouse monoclonal IgG2a SMA, 61001 (Progen, Heidelberg, Germany; 1 in 100), glucagon (also stains for GLP-1) rabbit polyclonal sc-13091 (Santa Cruz; 1 in 200), glucagon goat polyclonal sc-7782 (Santa Cruz; 1 in 100), Ki67 rabbit polyclonal ab15580 (Abcam, Cambridge, UK; 1 in 500), E-cadherin mouse monoclonal Ab76055 (Abcam, Cambridge, UK), GFP goat polyclonal Ab5450 (Abcam, Cambridge) and Vimentin rabbit monoclonal Ab92547 (Abcam, Cambridge). Mouse antibodies were used in conjunction with a mouse on mouse kit BMK-2202 (Vector, Peterborough, UK). Following this, slides were washed 3×10 minutes in PBS and incubated with fluorescent secondary antibody, anti-rabbit IgG (whole molecule), F(ab)2 fragment-Cy3 (Sigma-Aldrich) for 1 hour at room temperature. Negative controls included a PBS negative control, a rabbit serum control and frozen sections from mice lacking RFP. Image capture was performed on a Nipkov spinning disc confocal microscope (Hodson, Rutter, JCI, 2013) using a 20× lens or a Zeiss LSM 510 confocal microscope. Fluorophores were excited using 491 nm and 561 nm laser lines. DAPI was visualised at 359 nm. Emitted light was captured using filters centred on 480/40 nm, 535/30 nm and 630/75 nm for DAPI, Alexa-Fluor-488 and Alexa-Fluor-568, respectively. Uniform linear adjustments were performed to contrast/brightness to improve image quality as required.

### Brain sections

Coronal sections were cut at 30 μm using a cryostat and washed in 0.1 M phosphate buffer (PB; pH 7.4). After incubation in blocking buffer containing 10% sheep serum, 0.1% Triton X-100 diluted in 0.1 M PB for 30 minutes at room temperature, sections were transferred to 1:1000 anti-DsRed (Clontech #632496) in blocking buffer and incubated overnight on a shaker at 4°C. Following primary antibody incubation, sections were washed 3×10 minutes in PB followed by incubation in 1:500 Cy3-conjugated anti-rabbit secondary antibody (Sigma #C2306) in blocking buffer for 2 hours on a shaker at room temperature. Sections were washed in PB, mounted onto slides and left to air dry before being coverslipped and viewed using epifluorescence (Nikon Eclipse 80; Kingston upon Thames, Surrey, United Kingdom). Photomicrographs were taken with a Micropublisher 3.3 RTV camera and QCapture Pro software (Qimaging Inc., Surrey, BC, Canada).

### Measurement of GLP-1 and enteroglucagon from tissues

GLP-1 and enteroglucagon peptide hormones were extracted by boiling the tissues for 15 minutes in 0.5 M acetic acid (Bryant et al., 1983). The resulting supernatant was assayed by radioimmunoassay using in-house RIA for enteroglucagon and GLP-1 (Bryant et al., 1983; Savage et al., 1985). Results are expressed as pmol per gram of wet tissue.

### Survival curves

Survival curves were constructed using GraphPad PRISM software 5.01. We assigned values of 1 to events of premature recorded death and 0 to events of death where the precise time of death was either not well defined or was unknown owing to planned terminal studies or euthanasia owing to ill health. Survival curves were compared using the Log-rank Mantel-Cox test.

### Statistics

Data was analysed using GraphPad PRISM software, version 5.01. Two-tailed, unpaired Student’s *t*-tests with Bonferroni correction and one-way and two-way ANOVA (normal, not repeated measures) were used to calculate *P*-values where appropriate. *P*-values ≤0.05 were considered significant. Data are expressed as mean±s.e.m. or mean±s.d.

## Supplementary Material

Supplementary Material
