# Supplementary material for: The Peutz-Jeghers kinase LKB1 suppresses polyp growth from intestinal cells of a proglucagon-expressing lineage in mice
Source: Dis Model Mech. 2014 Sep 4;7(11):1275–86. doi: 10.1242/dmm.014720 (PMC4213731; doi:10.1242/dmm.014720)
Supplement: Supplementary Material [file supp_7_11_1275__index.html]

The Peutz-Jeghers kinase LKB1 suppresses polyp growth from intestinal cells of a proglucagon-expressing lineage in mice — Supplementary Material 

# The Peutz-Jeghers kinase LKB1 suppresses polyp growth from intestinal cells of a proglucagon-expressing lineage in mice

## DMM014720 Supplementary Material

**Files in this Data Supplement:**

- **Supplementary Material**
