# Supplementary material for: The Peutz-Jeghers kinase LKB1 suppresses polyp growth from intestinal cells of a proglucagon-expressing lineage in mice
Source: Dis Model Mech. 2014 Sep 4;7(11):1275–86. doi: 10.1242/dmm.014720 (PMC4213731; doi:10.1242/dmm.014720)
Supplement: Supplementary Material [file supp_7.11.1275_DMM014720.pdf]

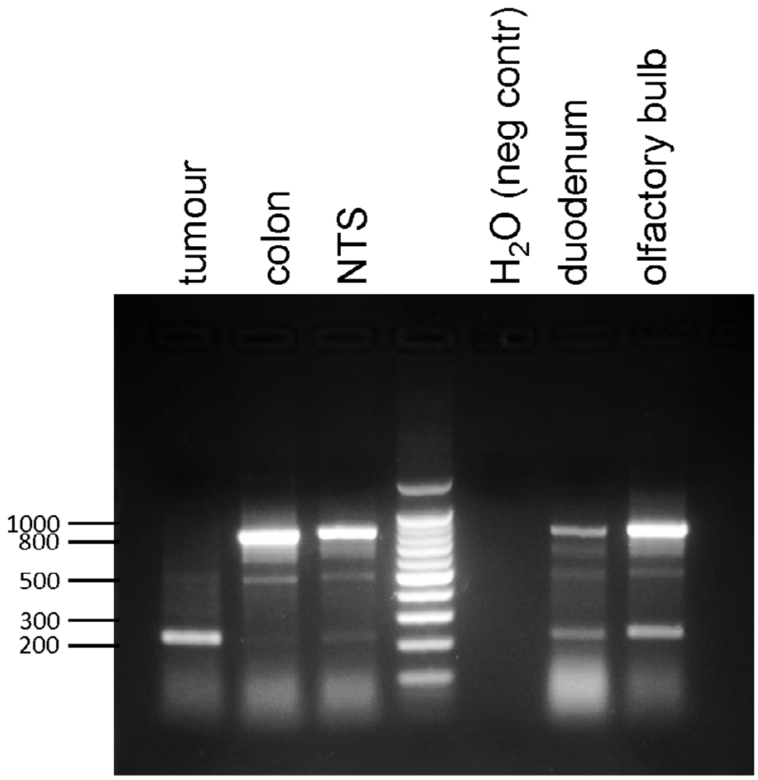

Supp. Figure 1. RT-PCR analysis of LKB1 gene recombination in different tissues from GluLKB1KO mice. Product sizes were 864 and 228 bp for flox'd and null alleles, respectively. As expected, the bands for the flox'd allele were much stronger in most tissues, reflecting the fact that Lkb1 is expressed in many cell types beside those where the proglucagon promoter is active. The null allele is found in all tissues tested that contain cells with proglucagon promoter activity. The relative paucity of the band reflects the fact that these cells constitute just a small fraction of these tissues. The molecular weight ladder shows bands at 100 to 1000 bp at 100 bp steps and an additional 1500 bp band. The strongest band indicates 500 bp.

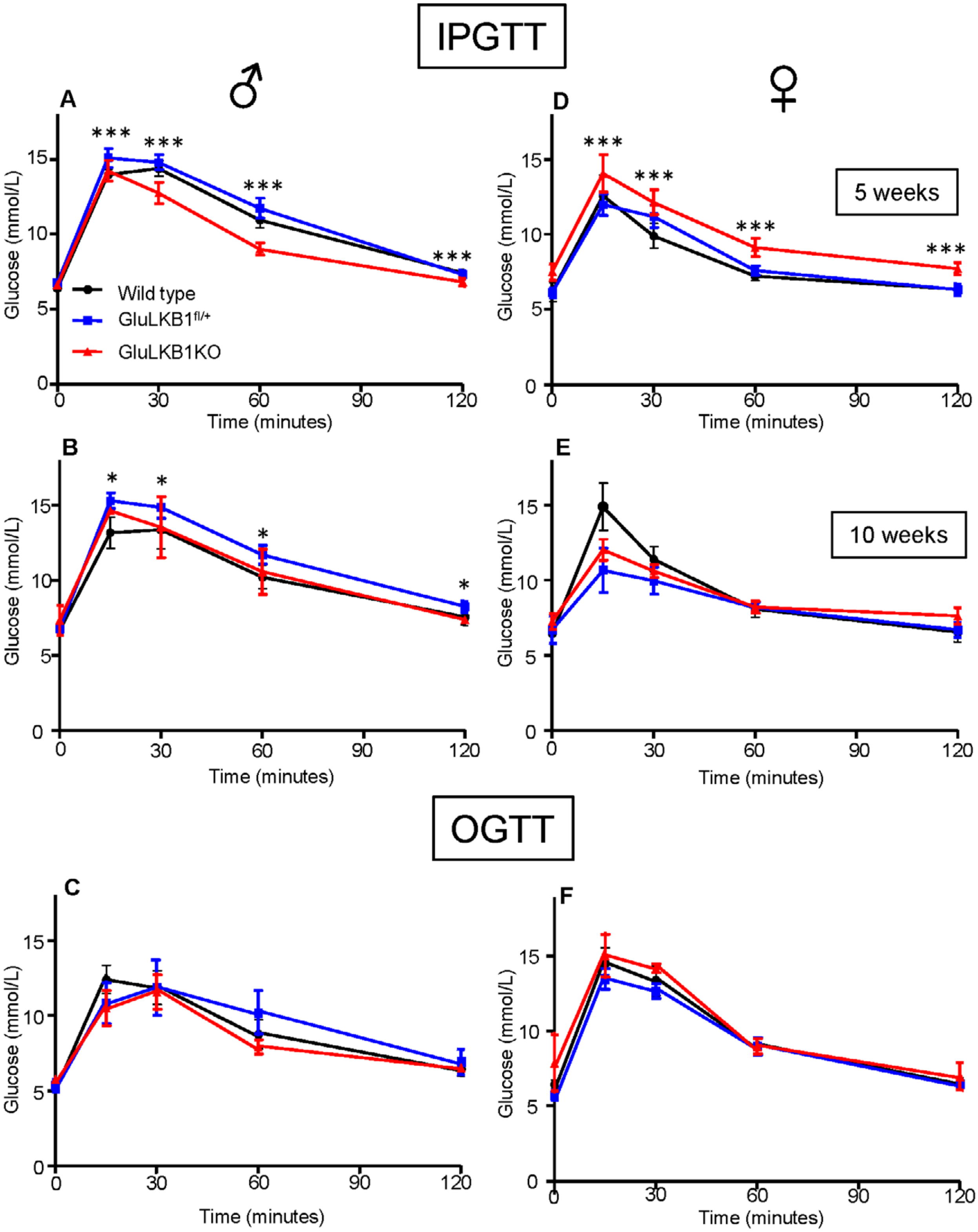

Supp. Figure 2. Glucose metabolism in control and GluCre-mediated LKB1 deletion mice. (A-E) Intraperitoneal glucose tolerance tests performed at 5 weeks and 10 weeks in male (A, B) and female (D, E) mice. (C, F) Oral glucose tolerance tests performed in male (C) and female (F) mice. Analysis of curves was by two way ANOVA. There was a significant difference between male wild type, GluLKB1fl/+ and GluLKB1KO mice in the IPGTT at 5 ($P < 0.0005$, ***) and 10 weeks (P=<0.05, *) and also for the females at 5 weeks (P=<0.001, ***). For the OGTT, no difference was seen between the groups for the males or females at 10 weeks. Subgroup analysis comparing wild type to GluLKB1KO mice failed to find a significant difference between these groups. Data are represented as mean ± SEM; n = 3-9.

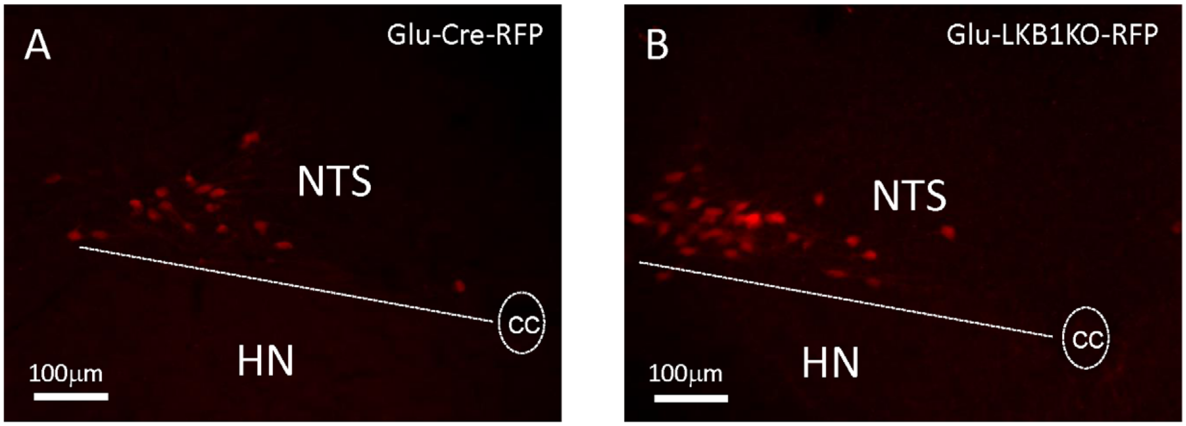

Supp. Figure 3. Distribution and morphology of GLP-1 neurons in hindbrain of control and GluCre-mediated LKB1 deletion mice. Photomicrographs showing RFP-immunoreactive GLP-1 neurons (red) in the nucleus tractus solitarius (NTS) of control mice (A) and GluLKB1KO mice (B). The dashed lines indicate the border to the hypoglossal nucleus (HN) and outline the central canal (cc) as landmarks.

Supp. Table 1. The sequences of primers used for RT-PCR Cyclophilin, Proglucagon, Cre, LKB1 and tdRFP.

**Gene**	**Forward primer**	**Reverse primer**
**Cyclophilin**	TATCTGCACTGCCAAGACTGA	CCACAATGCTCATGCCTTCTTTCA
**Proglucagon**	ATACCGCAAAGAGCACGAGAAG	CTCAAGAGCAGCGAAAGCGTCACAG
**Cre**	ATGTCCAATTTACTGACCG	CGCCGCATAACCAGTGAAAC
**LKB1**	GGGCTTCCACCTGGTGCCAGCCTGT	GAGATGGGTACCAGGAGTTGGGGCT
**tdRFP**	CTACAGGAACAGGTGGTGG	CTGTTCCTGGGGCATGGC

Supplementary Table 1. Primers used for RT-PCR
